# Endoscopic percutaneous repair of laryngeal cleft

**DOI:** 10.3389/fped.2023.1113894

**Published:** 2023-02-23

**Authors:** XinYe Tang, Yang Yang, ZhiHai Zhang, Rong Sun

**Affiliations:** ^1^ Department of Otolaryngology, Children's Hospital of Chongqing Medical University, Chongqing, China; ^2^ Ministry of Education Key Laboratory of Child Development and Disorders, Chongqing, China; ^3^ National Clinical Research Center for Child Health and Disorders, Chongqing, China; ^4^ China International Science and Technology Cooperation Base of Child Development and Critical Disorders, Chongqing, China; ^5^ Department of Physical Examination, The First Affiliated Hospital of Chongqing Medical University, Chongqing, China

**Keywords:** laryngeal cleft, aspiration, dyspnea, endoscopic, repair

## Abstract

**Objective:**

The aim of this study was to describe a novel surgical technique of endoscopic percutaneous repair in pediatric patients with type 1, type 2 and type 3 laryngeal cleft (LC).

**Methods:**

A retrospective study involving 12 patients with LC was performed at a tertiary pediatric hospital between February 2021 and June 2022. Endoscopic percutaneous repair was performed in all the patients. Information such as demographics, comorbidities, history of tracheostomy and the open approach for the repair, type of cleft and complications were analyzed.

**Results:**

Twelve patients were diagnosed with LC. The median age of the patients at the time of surgery was 8.50 months (interquartile range, 49.50 months). Seven patients had tracheomalacia, four patients had subglottic stenosis, three patients had laryngomalacia. No surgical complications occurred in the 10 patients who underwent the primary procedure. For two patients who underwent a secondary procedure, endoscopic percutaneous repair failed again to heal the cleft. During the follow-up period after surgery, none of the patients had stridor, recurrent pneumonia, feeding difficulties, or dyspnea. Follow-up modified barium swallow postoperatively demonstrated no aspiration in 10 patients. Only the 2 patients with a secondary procedure had intermittent cough while taking large gulps of water. The cure rate of endoscopic percutaneous repairer was 83.3% (95% confidence interval: 73.9%–92.8%).

**Conclusion:**

Endoscopic percutaneous repair should be considered as an alternative to the open transcervical approach and the traditional endoscopic approach for type 1, type 2 and type 3 LC.

## Introduction

Laryngeal clefts (LC) are rare congenital airway anomalies that were charactered by a craniocaudal fissure in the separation between the laryngotracheal airway and pharyngoesophageal tract. LC were first reported by Richter in 1792 (1). Symptoms include aspiration, recurrent pneumonia and feeding difficulties. The Benjamin and Inglis classification is the most widely used system to describe LCs as follows: type 1, interarytenoid defect; type 2, partial defect in the cricoid cartilage; type 3, complete defect of the cricoid cartilage with or without cervical tracheal involvement; and type 4, extending into the thoracic trachea (2). For type 1, type 2 and most type 3 LC, the endoscopic repair is the preferred option (3–6). However, repairing using traditional endoscopic approaches, especially for type 3 LC, is difficult. For some type 3 LC, the open transcervical approach for the repair is generally recommended (7, 8). But the anterior midline incision of the larynx and trachea may affect the stability of the airway structure (4, 5). To address this issue, we were inspired by the endoscopic percutaneous suture lateralization for neonatal bilateral vocal folds described by Montague et al. (9), in which one end of the suture was pierced percutaneously through the airway by a 22-gauge needle and then removed from the skin using another tractive suture. Similarly, we hypothesized that if one end of the suture was pierced percutaneously through the airway and the esophagus and then pulled out by another tractive suture, “simple suture procedures of LC” could be achieved. Herein, we describe a novel technique for endoscopic percutaneous repair of type 1, Type 2 and type 3 LC.

## Materials and methods

The present study retrospectively reviewed the medical records of patients who underwent endoscopic percutaneous repair of LC at the Children's Hospital of Chongqing Medical University between February 2021 and June 2022. Their data were analyzed from February 2022 to July 2022. Information on age, sex, gestational age, comorbidities, history of tracheostomy, open approach for repair, type of cleft, main symptoms, and complications was collected. The extent of the cleft was examined during the operation. The Institutional Review Board of Chongqing Medical University approved the study protocol. The study was complied with the Helsinki Declaration of 1975 (revised in 2008).

All the patients underwent endoscopic percutaneous repair of LC. Surgical repair was performed under general intravenous anesthesia using propofol and spontaneous respiration. The vocal cords are also anesthetized with topical lidocaine (2 mg/kg). All operations were performed under a Benjamin-Holinger (Storz) laryngoscope and 4 mm 0° telescope.

**Figure 1:**
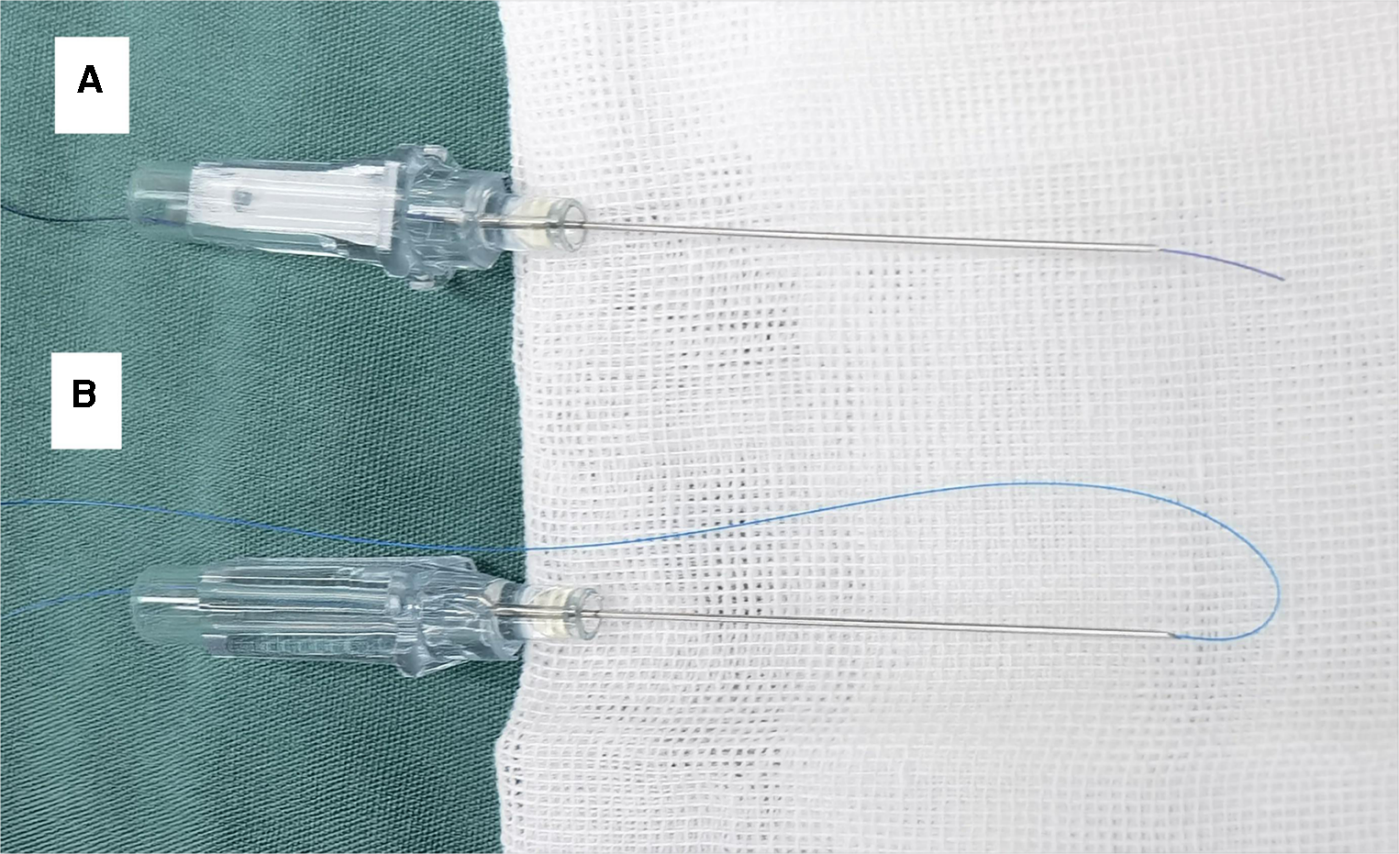
Needle and suture were prepared: (**A**) suture #1 (4-0 medtronic absorbable suture) was placed in a 22-gauge indwelling needle, and the tip of the suture #1 was slightly exposed to the bevel of the needle; (**B**) suture #2 (5–0 prolene suture) was placed in a 22-gauge indwelling needle, and a loop was formed at the front end.

First, needle and suture were prepared. A single strand of 4-0 or 5-0 Medtronic absorbable suture (suture #1) was placed in a 22-gauge indwelling needle (BD). The tip of the suture #1 was slightly exposed to the bevel of the needle (Figure 1A). A single strand of 5-0 Prolene suture (suture #2) was placed in a 22-gauge indwelling needle. Through the bevel of the needle, suture #2 was folded back into two strands, and a loop was formed at the front end (Figure 1B).

**Figure 2:**
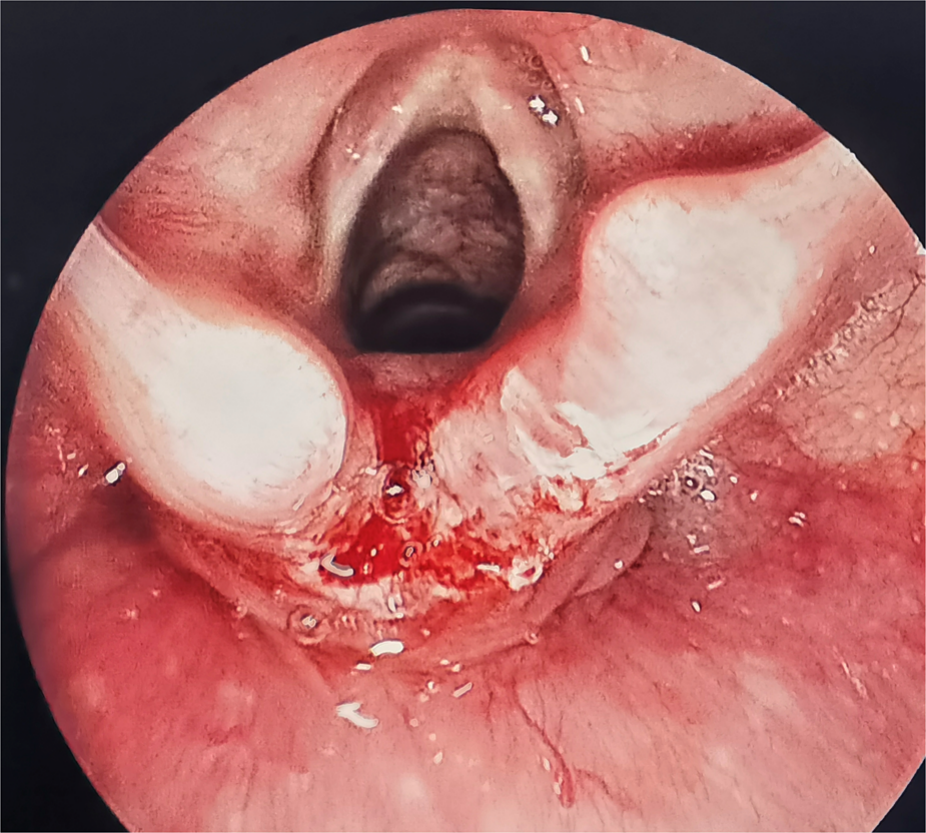
A fresh incision at the mucosal border of the cleft was made.

Second, fresh wounds at the free edge of the laryngeal fissure mucosa were created. The edge and apex of the cleft were then denuded by needle-tip electrocautery (Storz) (10). Subsequently, a normal saline cotton ball was used to wipe the wound surface to make fresh wound (Figure 2).

**Figure 3:**
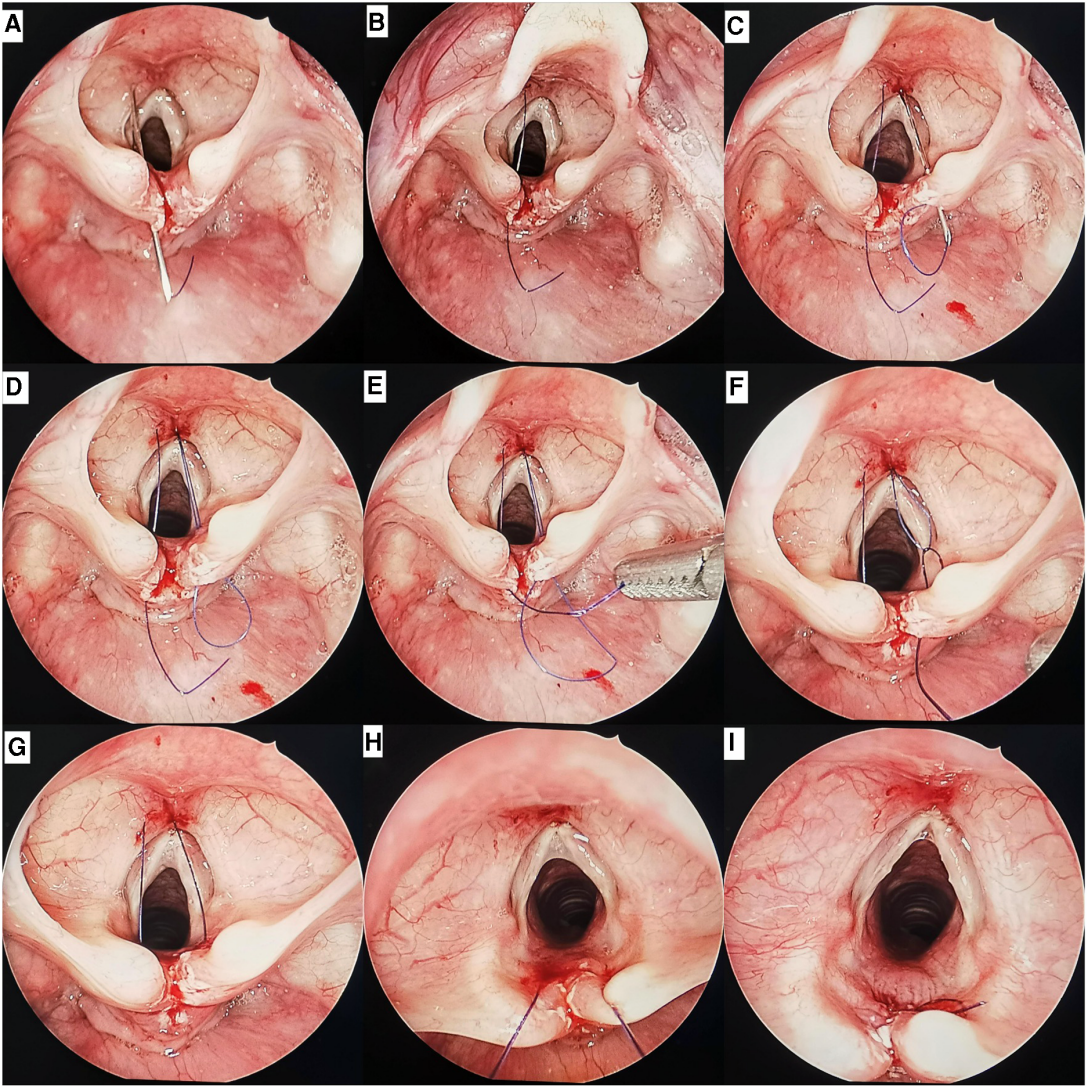
Endoscopic views of a type 1 cleft by endoscopic percutaneous repair: (**A**) the 22-gauge needle with suture #1 inserted into the airway percutaneously, then penetrated the mucosal at the left edge of the cleft and entered the esophagus. (**B**) The end of suture #1 was pulled into the esophagus, the 22-gauge needle was withdrawn. (**C**) The 22-gauge needle with suture #2 inserted into the airway percutaneously, then penetrated the mucosal at the right edge of the cleft and entered the esophagus. (**D**) The loop of suture #2 was pulled into the esophagus, the 22-gauge needle was withdrawn. (**E**) The end of suture #1 was passed though the loop of suture #2. (**F**) The end of suture #1 was pulled into the airway by the loop of suture #2. (**G**) Both ends of suture #1 were located in the airway. (**H**) The both ends of suture #1 was pulled out of the barrel. (**I**) The knot was tied on the side of the airway.

Third, cleft was closed percutaneously. A 22-gauge needle with suture #1 was placed percutaneously within about 3 mm to the left of the midline of the neck. After entering the airway, the needle tip then penetrated the mucosal at the left edge of the cleft (approximately 0.5 mm from the needle insertion point to the free edge of cleft) and entered the esophagus (Figure 3A). The single-strand suture #1 was then drawn into the esophagus with laryngeal forceps, and the 22-gauge needle was withdrawn from the esophagus and airway (Figure 3B). Similarly, another 22-gauge needle with suture #2 was placed percutaneously in the esophagus from the right side (Figure 3C). The loop of suture #2 was drawn into the esophagus, and the 22-gauge needle was withdrawn (Figure 3D). The end of suture #1 was passed though the loop of suture #2 (Figure 3E). The loop of suture #2 was then drawn into the airway with the end of suture #1 (Figure 3F). After suture #2 is removed, both ends of suture #1 in the airway are pulled out of the barrel of the Benjamin-Holinger laryngoscope (Figures 3G–H). When tying the knots, a knot pusher (Storz) was routinely used. The knot was tied to the side of the airway (Figure 3I). The cleft was closed, from the caudal to the cranial part, with a single layer. Approximately 1–6 sutures were used depending on the extent of the cleft.

After the operation, three patients with type 3 LC without tracheotomy were monitored in the pediatric intensive care unit for 3 days. The remaining patients return directly to the general ward postoperatively. Antibiotics were intravenously administered for 7 days. Systemic glucocorticoids (dexamethasone 0.5 mg/kg) were administered for 3 days. The patients were fed using a gastric tube immediately postoperatively and were switched to oral feeding 4 weeks later. Proton pump inhibitors were routinely administered for at least 4 weeks. All the patients underwent flexible fiberoptic laryngoscopy to assess the surgical incision 1 weeks postoperatively. Modified barium swallow (MBS) was performed to evaluate postoperative swallow function 3 months postoperatively. Descriptive statistics were used to analyze the overall group. All data are presented as medians and interquartile ranges (IQRs) for non-normally distributed continuous data. Categorical data are presented as percentages.

## Results

A total of 12 patients (6 male and 6 female) who had LC were included in the present study (Table 1). The main symptoms were difficulties during feeding in 7 patients, recurrent pneumonia in 3 patients, stridor in 4 patients and dyspnea in 3 patients with type 3 LC. Congenital anomalies were identified in 6 patients (50%); 4 patients had subglottic stenosis; 3 patients had VACTERL (vertebral, anal, cardiac, tracheal, esophageal, renal, and limb anomalies) association; and 1 patient had congenital tracheal stenoses. According to the Myer-Cotton classification system, the 4 patients with subglottic stenosis were all grade I (0%–50% obstruction of subglottic airway) (11). The associated comorbidities were observed in 10 patients (83%): 7 patients had tracheomalacia, 3 patients had laryngomalacia, 2 patients had pharyngomalacia, 2 patients had bronchiectasis and atelectasis, and 2 patients had gastroesophageal reflux disease. According to the Benjamin–Inglis classification system (2), 5 patients had type 1 LC, 2 patients had type 2 LC, 5 patients had type 3 LC, and none of the patients had type 4 LC. In 3 patients with type 3 LC, the cleft extended through the cricoid cartilage and ended above the first tracheal ring. In 2 patients with type 3 LC, the cleft passed to the first tracheal ring and ended above the second tracheal ring (Table 2). The median age of the patients at the time of surgery was 8.50 months (IQR, 49.50 months). Of the 12 patients treated using the endoscopic percutaneous approach, 10 patients (83%) were treated with a primary procedure and 2 patients (17%) with a secondary procedure after a previous surgery using an open transcervical approach with tracheostomy. The median operative time of endoscopic percutaneous repair in the series was 89.00 min (IQR, 51.25 min). No surgical complications occurred in any of the patients who underwent a primary procedure. For 2 patients who underwent a secondary procedure, endoscopic percutaneous repair failed again to heal the cleft. The cleft was repaired using the second open transcervical approach. The tracheostomy tube was successfully removed 3 months postoperatively. During the follow-up period, none of the patients had stridor, recurrent pneumonia, feeding difficulties, or dyspnea postoperatively. All the patients had aspiration of nectar-thick liquids, as documented by MBS before the repair. Follow-up MBS postoperatively indicated no aspiration in 10 patients. Only the 2 patients who underwent a secondary procedure had intermittent cough while taking in large gulps of water. The cure rate of endoscopic percutaneous repair was 83.3% (95% confidence interval: 73.9%-92.8%).

**Table 1:** Characteristics of patients with laryngeal clefts.

Sex/Age at new method, mo	Major Symptoms	Syndrome or Congenital anomalies	Comorbidities	Cleft type
M/3	Stridor	None	Laryngomalacia	Type 1
F/3	Choking and coughing during feeding	SGS (Grade I)	GERD	Type 1
			Tracheomalacia	
M/7	Choking and coughing during feeding	None	Severe malnutrition	Type 1
F/81	recurrent pneumonia	CTS	None	Type 1
F/17	Stridor	VACTERL	Tracheomalacia	Type 1
	Choking and coughing during feeding	SGS (Grade I)	Pharyngomalacia	
M/61	Choking and coughing during feeding	VACTERL	Tracheomalacia	Type 2
		SGS (Grade I)		
F/9	Stridor	SGS (Grade I)	None	Type 2
	Choking and coughing during feeding			
M/8	Stridor	None	GERD	Type 3
	Choking and coughing during feeding		Laryngomalacia	
M/1	Choking and coughing during feeding	None	Tracheomalacia	Type 3
M/57	Dyspnoea	None	Bronchiectasis	Type 3
	Recurrent pneumonia		Atelectasis	
			Tracheomalacia	
F/43	Dyspnoea	None	Bronchiectasis	Type 3
	Recurrent pneumonia		Atelectasis	
			Tracheomalacia	
F/7	Dyspnoea	VACTERL	Tracheomalacia Laryngomalacia Pharyngomalacia	Type 3

CTS, congenital tracheal stenoses; GERD, gastroesophageal reflux disease; SGS, subglottic stenosis; VACTERL, vertebral, anal, cardiac, tracheal, esophageal, renal and limb anomalies.

**Table 2:** Endoscopic percutaneous repair and follow-up.

Cleft type	Extent of the cleft	Operative time, min	Number of stitches	MBS after repair	Follow-up, mo
Type 1	Interarytenoid cartilage	40	1	No aspiration	6
Type 1	Interarytenoid cartilage	80	1	No aspiration	7
Type 1	Interarytenoid cartilage	60	1	No aspiration	8
Type 1	Interarytenoid cartilage	65	1	No aspiration	6
Type 1	Interarytenoid cartilage	83	1	No aspiration	3
Type 2	Partial cricoid cartilage	75	3	No aspiration	13
Type 2	Partial cricoid cartilage	95	3	No aspiration	5
Type 3	`Above first tracheal ring	115	2	No aspiration	16
Type 3	Above first tracheal ring	145	3	No aspiration	13
Type 3	Above Second tracheal ring	175	6	No aspiration with puree and honey thick	8
Type 3	Above Second tracheal ring	120	3	No aspiration with puree and honey thick	8
Type 3	Above first tracheal ring	115	3	No aspiration	3

MBS, modified barium swallow.

## Discussion

The present study demonstrated that endoscopic percutaneous repair of the LC is a feasible technique. During the surgery, the cleft was successfully closed in all patients, and no surgical complications occurred. Apart from two patients who required open transcervical repair, none of the other patients had secondary dehiscence.

Endoscopic percutaneous repair has clear advantages over open transcervical surgery. Firstly, anterior incision of the larynx and trachea was avoided, thus reducing the potential risk of laryngotracheal frame instability (4, 5, 12). Secondly, the endoscopic percutaneous approach may avoid postoperative tracheal intubation or tracheostomy (4, 13, 14). Lastly, some of the risks associated with the open transcervical approach, including recurrent laryngeal nerve injury, neck infection, and pharyngeal fistula, are rare (5).

In our experience, the deepest part of the type 3 LC has insufficient space, and repair of this part using the traditional endoscopic approach is very difficult. To ensure operability of the procedure, a smaller suture needle, such as a 6-0 Vicryl suture on a *P*-1 needle, was selected (4, 5). The smaller suture needle is likely to be completely submerged into the tissue, and the needle cannot be released. Moreover, the needle holder cannot hold the suture needle firmly, which may easily cause it to fall off. Therefore, less tissue is sutured at the edge of the cleft, which may increase the risk of secondary dehiscence after traditional endoscopic repair. Endoscopic percutaneous repair can solve the aforementioned problems with the traditional endoscopic approach. The 22-gauge indwelling needle can be freely moved in and out of the larynx and trachea, ensuring that as much tissue as possible is sutured to both sides of the cleft. This increases the strength of the wound-healing scar tissue. Therefore, endoscopic percutaneous repair for type 3 LC has better advantages over the traditional endoscopic approach.

The endoscopic percutaneous approach depends on good endoscopic exposure. The lateral edges and distal end of the cleft must be visible and accessible for needle placement. For patients with the Pierre Robin syndrome, Treacher Collins syndrome, or Down syndrome, endoscopic exposure may be difficult (6, 15, 16), and an open approach needs to be considered.

In our series two of the five children with type 3 LC underwent three separate repairs before closure was successful. The initial management of the two children was through tracheotomy and transcervical repair. The mucosa at the lateral edges of the cleft was thin and brittle, which may interfere with healing after endoscopic percutaneous approach. The cleft was successfully repaired by second transcervical approach with costal cartilage grafting. In the second transcervical operation, the esophageal mucosa at the edge of the cleft was sufficiently dissociated to ensure that the thick and healthy mucosal tissue was sutured. Koltai et al. have reported that one child had two previous transcervical repairs of the type 2 LC that left the interarytenoid tissues thin, friable, and covered with granulations (17). Therefore, endoscopic percutaneous repair should be carefully considered for children with type 3 LC who have undergone repeated operations.

In our study, tracheomalacia, subglottic stenosis, and laryngomalacia were the most common airway comorbidities and anomalies. Seven of 12 patients (58%) had tracheomalacia. These tracheomalacia cases were mild and did not result in severe dyspnea. Our results demonstrated that the mild tracheomalacia did not have a significant effect on the repair of LC. For severe tracheomalacia, which results in severe dyspnea, tracheotomy may be required to create a safe airway before the repair of LC (5). A very strong correlation between LC and subglottic stenosis was identified. A previous study has reported that 26% of patients with LC have subglottic stenosis (18). Four of 12 patients (33%) in our series had subglottic stenosis, which were all grade I. Our results demonstrated that the grade I subglottic stenosis did not affect the success rate of LC repair in our series of patients. For the patients with LC and severe subglottic stenosis (grade II-IV), endoscopic percutaneous repair is not recommended. First, the airway is bound to become narrow after LC is closed (4). Second, endoscopic airway surgery will inevitably lead to varying degrees of mucosal edema (4). One millimeter of annular edema at the subglottic level reduces the cross-sectional area by nearly 60% (19). All of the abovementioned factors can lead to postoperative airway stenosis and long-term tube placement in patients with LC and severe subglottic stenosis. Therefore, we recommend open surgery with costal cartilage grafting for patients with LC and severe subglottic stenosis (20–22). Laryngomalacia is the common associated anomaly in LC (23). Three patients (25%) in our series had laryngomalacia. The upper glottic airway narrowed after LC closure. The aryepiglottic folds were divided to expand the airway during LC repair (24).

In conclusion, endoscopic percutaneous repair with the open transcervical approach significantly reduces perioperative and postoperative morbidity. Endoscopic percutaneous repair has better maneuverability and firmer sutures for type 3 LC than the traditional endoscopic approach. Sufficient exposure and a healthy mucosal structure at the lateral edges of the cleft are the major factors that should be considered when determining whether a patient is a candidate for endoscopic percutaneous repair. For the patients with LC and severe subglottic stenosis (grade II-IV), we recommend open repair rather than endoscopic percutaneous repair. Endoscopic percutaneous repair may be a suitable technique for the treatment of type 1, type 2, and type 3 LC.

## Data Availability

The original contributions presented in the study are included in the article/Supplementary Material, further inquiries can be directed to the corresponding author/s.
